# Validation of reliability and validity of the Chinese version of the active-empathic listening scale

**DOI:** 10.3389/fpsyt.2022.938461

**Published:** 2022-08-23

**Authors:** Hang Guo, Lemin Lin, Ziming Jia, Jiaying Sun, Zisen Zhuang, Lisa Duan, Jiangnan Sun

**Affiliations:** ^1^Postgraduate Group, Logistics University of People’s Armed Police Force, Tianjin, China; ^2^The Health Team of the Fifth Mobile Detachment of the First Mobile Corps of the Armed Police Force, Dingzhou, China; ^3^College of Field Engineering, Army Engineering University of People’s Liberation Army of China, Nanjing, China; ^4^Department of Psychology, The First Affiliated Hospital of Fujian Medical University, Fuzhou, China; ^5^Institute for Military Psychological Efficacy Evaluation and Stress Intervention, Characteristic Medical Center of Chinese People’s Armed Police Force, Tianjin, China

**Keywords:** active-empathic listening scale (AELS), active listening, reliability, validity, Chinese version

## Abstract

**Background:**

Active-empathic listening (AEL) is the active and emotional involvement of a listener that can take place in at least three key stages of the listening process. Bodie has developed and validated a self-reported, 11-item, three-factor active-empathic listening scale (AELS) in English with good reliability (Cronbach’s alpha = 0.86) to assess AEL abilities. Nevertheless, a Chinese version of the AELS had not been established and validated yet.

**Objective:**

The objective of the present study was to examine the reliability and validity of the Chinese version of the AELS.

**Methods:**

After translating the scale into the Chinese version, 834 college students completed the test. After 4 weeks, 206 participants were tested again on the Chinese AELS to examine retest reliability. The critical ratio method and the item-total correlations were used for the item analysis. Exploratory factor analysis (EFA) and confirmatory factor analysis (CFA) were performed to examine the construct validity. The internal consistency of the scale was analyzed with Cronbach’s alpha and McDonald’s Omega. Interclass correlation coefficient (ICC) was used to examine the scale’s retest reliability. The Interpersonal Reactivity Index (IRI) was used to examine the convergent validity. Pearson correlation analysis was conducted.

**Results:**

Each item of the Chinese AELS had a good discrimination, and the item-total correlation of each item ranged from 0.51 to 0.73. EFA extracted three factors with characteristic root values greater than 1, which could explain 70.72% of the total variance. CFA indicated an adequate fit of the three-factor model (χ^2^/df = 2.250, root mean square error of approximation [RMSEA] = 0.055, the comparative fit index [CFI] = 0.971, the Tucker-Lewis index [TLI] = 0.959, and the goodness of fit index [GFI] = 0.959). The internal consistency reliability was acceptable (sensing: α = 0.79/ω = 0.78, processing: α = 0.83/ω = 0.83, responding: α = 0.79/ω = 0.79, and AELS: α = 0.87/ω = 0.87). Retest reliability of the scale at 4-week intervals by an ICC was 0.563. The Chinese AELS was significantly correlated with each dimension of IRI.

**Conclusion:**

The reliability and validity of the Chinese AELS met the basic psychometrics requirements. Therefore, the scale can be potentially used to assess the active empathic listening abilities of people in China.

## Introduction

The concept of “active listening” put forward by Rogers is one of the most representatives of humanistic therapy. It has been widely used in various fields, such as in psychological counseling, educational (1), medical (2), and occupational settings (3–5). It was described as a process that includes techniques, such as maintaining eye contact, not interrupting the speaker, making encouraging comments or non-verbal gestures, formulating appropriate questions, paraphrasing, and summarizing in order to show a full understanding of the things said (6).

Active listening plays an important role for health care workers serving in the mental health field. A study, in 2017, aimed to understand the predictors of self-reported mental and emotional health among older African American men revealed that older African American men who reported that their doctors “never listen” to them were over two times as likely as their counterparts to experience downheartedness most or all of the time (7). In line with this finding, consistent data indicate that when patients perceived that their physicians listened more and were being more empathetic, they reported higher satisfaction with health care experiences and felt more supported (8). However, there is still a lack of instruments to measure the active listening ability of medical staff in China. In fact, research on “active listening” across the academic landscape has recognized the need to include empathy in its conceptualization (9).

Active-empathic listening (AEL) is the active and emotional involvement of a listener that can take place in at least three key stages of the listening process. Sensing describes a listener’s ability to understand relational aspects of speech. Processing is the cognitive aspect of listening, which involves attending to, comprehending, receiving, and interpreting messages. Responding describes the behavioral output of listening, i.e., verbal and non-verbal feedback. It was originally defined as a form of listening employed by salespeople, where customary active listening is merged with empathy to realize a “higher form of listening” (10). To assess effective vs. ineffective listening from the points of view of customers, Drollinger et al. developed the active-empathic listening scale (AELS) by referring to the previous scales that measured empathy (11) and active listening (12). In, Bodie refined this 11-item scale and adapted it to a more general social context, which is known as the AELS (13). Validity and temporal stability of the AELS have been demonstrated in a sample of United States undergraduate students from the Department of Communication Studies (9). The Greek and Japanese versions of AELS have been successfully developed. In (14), Kourmousi demonstrated the three-factor model and good reliability of the Greek version of the AELS in a Nationwide Sample of Greek Educators. In, Asai recruited 728 university students from five classes at two different universities. Participants were asked to complete the Japanese AELS and 59 individuals among them were administered again after 3 weeks to determine retest reliability. The results supported the three-factor model of the scale and demonstrated that the Japanese AELS has good internal consistency and moderate test-retest reliability (15, 16).

Researchers have studied the relationship of AEL with other variables in a variety of contexts. Bodie and Jones used an other-report version of the AELS and found that AEL was a crucial part of supportive communication (17). Pence and Vickery examined AEL in regard to emotional intelligence (EI) and personality, showing that EI predicted each AELS dimension. In addition, they found that there was a small, negative association between psychoticism in personality and the AELS subscales (18). Kourmousi surveyed Greek educators on their active empathic listening skills and identified enhancing factors of AEL (14). Eggenberger found out that AEL appears to be a significant predictor of academic achievement in the community college class (19). Furthermore, Brown et al. found a predictive effect of AEL on the professionalism in undergraduate occupational therapy students (20).

Although most measures of communicative competence or other social skills include items that tap elements of listening (13), a few of them directly assess AEL within all three stages of the listening process (sensing, processing, and responding). For instance, the Interaction Involvement Scale (IIS) includes a subscale labeled “perceptiveness” (e.g., during conversations I am sensitive to others’ subtle or hidden meanings). The Conversational Sensitivity Scale (CSS) includes subscales labeled “detecting meanings” (e.g., I often find myself detecting the purposes or goals of what people are saying in conversations) (9), which represents the “sensing” element in AEL. Furthermore, the Interpersonal Reactivity Index (IRI) emphasizes “empathy.” The IRI is a self-report scale that measures dispositional empathy and comprises the following four subscales: “personal distress (PS),” “empathic concern (EC),” “perspective taking (PT),” and “fantasy (FS) scale.” PS represents the tendency to experience distress and discomfort when observing the distress of others. EC denotes the tendency to experience feelings of other-oriented emotions, such as sympathy and compassion. PT signifies the extent to which one considers the point of view and feelings of others. The FS scale represents the tendency to imagine oneself in the place of fictional characters (11). Thus, the AELS shows superiority by simultaneously measuring three dimensions of AEL.

Currently, the AELS was mainly used in western countries, such as the United States, and a few studies on AEL have been carried out in China. The AELS is barely used among Chinese medical students who are future medical staff. An applicable Chinese AELS is needed for the Chinese cultural context. Therefore, the present study is aimed to translate the AELS into Chinese and validate its reliability and validity using a Chinese medical student sample.

## Materials and methods

### Participants and procedure

The present survey study was conducted from December 2021 to May 2022. A total of 956 medical students from two universities in Tianjin, China were included as volunteer participants. After excluding 85 individuals who did not complete the survey and 37 individuals who gave consistent answers, data from 834 participants were analyzed. After 4 weeks, 206 participants were randomly selected and retested on the Chinese AELS.

The survey was administered to student participants during class time after they were informed of the purpose of the study. They were also informed of the principle of voluntariness and confidentiality. In order to identify the retest participants, we used a number combination of the last two digits of their ID card no. and the last four digits of their mobile phone number. All the researchers had been trained before they recruited participants and administered the test.

### Measures

#### Chinese version of the active-empathic listening scale

The original AELS is an 11-item scale that measures active-empathic listening across three dimensions: sensing (4 items), processing (3 items), and responding (4 items). This scale is scored on a seven-point Likert-type scale ranging from 1 (never or almost never true) to 7 (always or almost always true). The total score is between 11 and 77. The higher the score, the stronger the AEL ability of the subject.

After being authorized by the scale’s original developer, Dr. Bodie, the English version of the AELS was then translated into Chinese. First, a psychologist and an English scholar translated the scale into Chinese, respectively. Then they held a discussion to compare two Chinese translations and determined whether the adopted words and items conveyed the same meaning, so they could form the preliminary Chinese version. Subsequently, another psychologist and another English scholar translated the preliminary version back into English, respectively. After discussion, they formed a back-translation version. Finally, two other bilingual experts in psychology were invited to perform a comparative analysis between the back-translated version and the original scale. The items with large differences were re-translated and translated back (21). This process was repeated two times. Dr. Bodie checked the back-translated version and provided feedback during the process. Integrating opinions from Dr. Bodie, the final version of the Chinese AELS was formed.

#### Chinese version of the Interpersonal Reactivity Index

Interpersonal Reactivity Index was used to verify the concurrent validity, which is an instrument for measuring empathy ability developed by Davis based on the multidimensional theory of empathy (11). Bodie reported a positive correlation between the AELS and the Empathic Responsiveness Scale (13). Thereafter, Asai demonstrated that the AELS was positively associated with IRI except the PS domain. Accordingly, the Chinese IRI was chosen to test the convergent validity.

The Chinese version of IRI (IRI-C) revised by Zhan is a 22-item self-report scale that includes four factors: PT, FS, EC, and PD. The scale uses a five-point Likert-type scale ranging from 1 (not true at all) to 5 (extremely true). Items 2, 5, 10, 11, and 14 are reverse scoring questions. The total score is between 22 and 110. Higher overall scores indicate greater empathy ability. Zhang et al. confirmed the validity of the IRI-C (22). In this study, the Cronbach’s α of IRI was 0.83.

### Statistical analysis

SPSS version 27.0 and AMOS version 24.0 were used for statistical analysis. The sample was randomly split into two groups, Group 1 (*n* = 417) for exploratory factor analysis (EFA) and Group 2 (*n* = 417) for confirmatory factor analysis (CFA). EFA and CFA were performed to verify the scale’s three-factor model. To assess the fit of the model, the chi-square (χ^2^), the comparative fit index (CFI), Tucker-Lewis index (TLI), the goodness of fit index (GFI), and the root mean square error of approximation (RMSEA) were calculated. The χ^2^/df < 3.00, RMSEA < 0.08, CFI > 0.90, TLI > 0.90, and GFI > 0.90 suggests a good fit (23). The internal consistency of the scale was analyzed with Cronbach’s alpha and McDonald’s Omega. Scales equal to or greater than 0.70 were considered satisfactory (15). Pearson correlation coefficients were used to explore the association among the subscales. The coefficient between 0.3 and 0.5 indicates moderate correlation and the coefficient over 0.5 indicates high correlation (15). The interclass correlation coefficient (ICC)was used to examine the scale’s retest reliability, and ICC ≥ 0.50 was considered acceptable (24). All statistical analyses used two-tailed tests. For all statistical evaluations, *p*-values less than 0.05 were considered indicative of significant differences.

## Results

### Item analysis

The survey analyzed a total of 834 participants (517 men and 317 women), with an average age of 21.11 ± 1.42 years. Mean scores for each item of the Chinese AELS are shown in Table 1. Results of the Item Analysis Critical ratio method and item-total correlations were used for the item analysis (25). Scores of 11 items were added to obtain the total score of the scale. The high group was made up of 27% of the highest scoring respondents (*n* = 232) while the low group was made up of 27% of the lowest scoring respondents (*n* = 236). An independent sample *t*-test was conducted and the results are summarized in Table 1.

**TABLE 1:** Results of the item analysis.

Item	Mean ± SD (*n* = 834)	Item-total correlation	High (*n* = 232)	Low (*n* = 236)	*t*	Sig.
1	4.41 ± 1.60	0.51**	5.55 ± 1.51	3.40 ± 1.09	−17.605	<0.001
2	4.47 ± 1.68	0.58**	5.71 ± 1.35	3.28 ± 1.19	−20.672	<0.001
3	4.81 ± 1.41	0.64**	5.86 ± 1.06	3.82 ± 1.39	−17.818	<0.001
4	4.57 ± 1.72	0.70**	5.98 ± 1.20	3.02 ± 1.26	−26.019	<0.001
5	4.35 ± 1.85	0.73**	5.86 ± 1.18	2.68 ± 1.61	−24.452	<0.001
6	4.33 ± 1.79	0.72**	5.85 ± 1.26	2.81 ± 1.48	−23.972	<0.001
7	4.52 ± 1.61	0.68**	5.84 ± 1.11	3.22 ± 1.47	−21.822	<0.001
8	4.88 ± 1.67	0.69**	6.14 ± 0.98	3.31 ± 1.52	−23.990	<0.001
9	5.03 ± 1.69	0.57**	6.09 ± 1.17	3.84 ± 1.70	−16.683	<0.001
10	4.89 ± 1.66	0.72**	6.17 ± 0.88	3.28 ± 1.40	−26.785	<0.001
11	5.19 ± 1.65	0.68**	6.33 ± 0.88	3.74 ± 1.74	−20.392	<0.001

**Correlation is significant at the 0.01 level (two-tailed).

Pearson correlation was used to analyze the correlation between item scores and total scores. The correlation coefficients ranged from 0.51 to 0.73 (*p* < 0.01), which indicates moderate to high correlations.

### Reliability

Cronbach’s alpha and McDonald’s Omega were adopted to test the internal consistency reliability of the scale and three factors. As presented in Table 2, internal consistency reliability is acceptable for sensing (α = 0.79/ω = 0.78), processing (α = 0.76/ω = 0.76), responding (α = 0.79/ω = 0.79), and total AELS scores (α = 0.86/ω = 0.85). After 4 weeks, 206 students were randomly selected to examine test-retest reliability. The test-retest analysis for the Chinese AELS measured by an ICC was 0.563 (95% CI 0.384–0.687).

**TABLE 2:** Internal consistency reliability of the scale.

Dimension	Cronbach’s α	McDonald’s Omega	Item number
Sensing	0.79	0.78	4
Processing	0.83	0.83	3
Responding	0.79	0.79	4
AELS	0.87	0.87	11

### Construct validity

**FIGURE 1:**
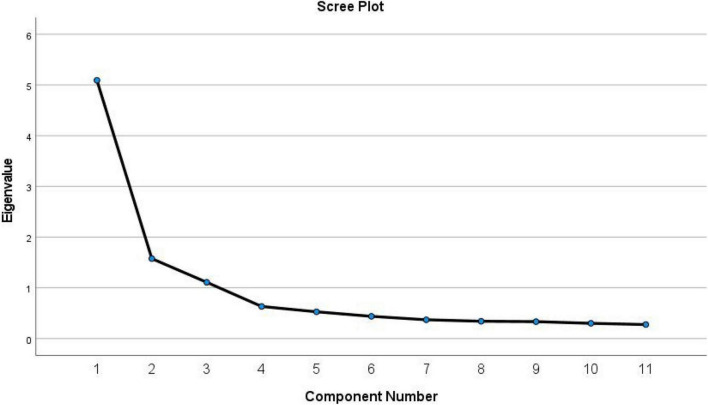
Screen plot of exploratory factor analysis for the Chinese version of the active-empathic listening scale (AELS).

Empathic listening scale was performed with the sample of Group 1 (*n* = 417) to identify the structure of the Chinese AELS. The Kaiser-Meyer-Olkin (KMO) coefficient was 0.876 and the Bartlett test of sphericity was significant (*χ^2^* = 2217.271; *p* < 0.001), which indicated the matrix is not an identity matrix and is appropriate for factor extraction. Principal component analysis and maximum variance rotation were used and extracted three factors (see Figure 1) with characteristic root values greater than 1, which could explain 70.72% of the total variance. The component loadings for each item are shown in Table 3.

**TABLE 3:** Factor loadings of exploratory factor analysis for the Chinese version of the active-empathic listening scale (AELS).

	Responding	Sensing	Processing
1	0.101	**0.840**	0.002
2	0.152	**0.847**	0.127
3	0.241	**0.729**	0.276
4	0.162	**0.653**	0.424
5	0.202	0.188	**0.831**
6	0.235	0.212	**0.819**
7	0.331	0.109	**0.799**
8	**0.810**	0.146	0.234
9	**0.829**	0.140	0.098
10	**0.724**	0.168	0.371
11	**0.699**	0.179	0.230

Bold indicates the highest factor loading for each item.

The CFA was performed with the sample of Group 2 (*n* = 417) to estimate if the three-factor model fitted the data well. As shown in Table 4, model fit indices for the model in the study results indicate an adequate fit of the three-factor model (*χ^2^*/df = 2.250, RMSEA = 0.055, CFI = 0.971, TLI = 0.959, and GFI = 0.959).

**TABLE 4:** Model fit indices for the model in the study.

Model	χ^2^/df	RMSEA	CFI	TLI	GFI
Three-factor model	2.250	0.055	0.971	0.959	0.959

Intercorrelations of AELS subscales were also calculated to provide further evidence of its construct validity, ranged from 0.460 to 0.606 (*p* < 0.01; see Table 5).

**TABLE 5:** Intercorrelations of active-empathic listening scale (AELS) subscales.

	Sensing	Processing	Responding	AELS
Sensing	1	0.460**	0.465**	0.778**
Processing		1	0.606**	0.822**
Responding			1	0.846**

** Correlation is significant at the 0.01 level (two-tailed).

### Convergent validity

The correlations between the Chinese AELS and the IRI are shown in Table 6. All dimensions of the AELS and the IRI were significantly correlated. The correlation coefficients ranged from *r* = 0.17 to *r* = 0.63 (*p* < 0.01).

**TABLE 6:** Correlations between the studied dimensions.

	Sensing	Processing	Responding	AELS
Perspective taking	0.39**	0.47**	0.42**	0.50**
Fantasy scale	0.42**	0.33**	0.32**	0.43**
Empathic concern	0.17**	0.38**	0.35**	0.36**
Personal distress	0.37**	0.30**	0.31**	0.39**
IRI	0.50**	0.55**	0.52**	0.63**

** Correlation is significant at the 0.01 level (two-tailed).

## Discussion

The presented study translated and examined the psychometric properties of the Chinese version of AELS in a sample of college students. Within this sample, the Chinese AELS showed good internal consistency reliability, construct validity, and convergent validity.

Item analysis showed a high correlation between each item and the total score, which were all significant, and the scores of high and low groups in each item were significantly different. These results suggested that all the 11 items on the scale had good discrimination.

Internal consistency reliability of the Chinese AELS estimated by Cronbach’s α and McDonald’s ω showed good reliability. Specifically, Cronbach’s α of the Chinese version was slightly lower than the Greek version (sensing = 0.82, processing = 0.76, responding = 0.82, and AELS = 0.87) (15) and was higher than the Japanese (sensing = 0.64, processing = 0.61, responding = 0.68, and AELS = 0.82) (16) and the original English version (sensing = 0.73, processing = 0.66, and responding = 0.78, AELS = 0.86) (13). McDonald’s ω of the Chinese version was higher than the Japanese (sensing = 0.72, processing = 0.62, responding = 0.77, and AELS = 0.86) (16). For the retest reliability of the scale, the ICC was used both in the Chinese and Japanese version, and the ICC of the Chinese version was slightly higher than the Japanese version (AELS total score = 0.51). Our result showed a moderate ICC, consistent with the Japanese version (16). While Bodie used Pearson correlation coefficients to evaluate the retest reliability in the original English version (*r* = 0.70) (9). Therefore, further testing in different populations is needed in the future to determine whether this scale does not exhibit good test-retest reliability for cultural reasons or the AELS itself has a moderate ICC.

Exploratory factor analysis extracted three factors with characteristic root values greater than 1. In addition, this model was confirmed by the CFA. With the EFA and CFA, our results again supported the three-factor structure of the AELS, consistent with the conclusions of the original scale and the Greek and Japanese versions. Moreover, the Chinese version and the English version had the same number of items in each subscale. Additionally, the three dimensions of the Chinese AELS significantly and positively correlated with each other. Moreover, each dimension was highly correlated with the overall score. Therefore, our findings indicated that the substructure of the Chinese version conformed to the theoretical conception of the original English version, and the scale had good construct validity.

The present study also provided further validity evidence by comparing the AELS to the IRI. The results supported good convergent validity of the Chinese AELS, as each dimension of AELS is significantly correlated with each dimension of IRI. Except for the low correlation between sensing and EC, all other correlation coefficients were medium or high. The reason may be that the sensing, which describes an active sensitivity to the emotional needs of a speaker and manifests in the listener attending to both the implicit and explicit aspects of others’ messages (9), is different from EC that investigates an individual’s emotional concern, warmth, and sympathy for others (22). It is worth noting that the personal distress of the Japanese IRI did not have a significant positive correlation with the Japanese AELS (16), which was different from the current study. This may be due to the specificity of the sample of medical students, who may have a greater ability to experience the pain of others.

## Limitations

In the present study, only the self-report version of the AELS was translated. According to Dr. Bodie, the other-report version can also be created by changing “I” to some other prompt, such as “My friend” or “My conversational partner” and then adjusting the verb tense (26). In addition, self-reporting data may contain a bias of participants. Therefore, research will be needed to further determine the validity of the other-report Chinese AELS. Moreover, due to the impact of the COVID-19 pandemic, participants were all from one college, which made the sample relatively lack representativeness. In the future, the scale can be further validated in a richer sample population so that the results will be more generalizable.

## Conclusion

The results of the current study indicate the good reliability and validity of the Chinese AELS, and it is suitable for the Chinese cultural context. In the future, this scale can be utilized to assess people’s active empathic listening abilities in China.

## Data availability statement

The raw data supporting the conclusions of this article will be made available by the authors, without undue reservation.

## Author contributions

HG and LL identified research content, researched route design, translated the scale, collected data, and contributed to data analysis and draft writing. ZJ and JYS contributed to data collection and entry. ZZ reviewed the draft and provided statistical guidance. JNS and LD contributed to the study route design, provided resources, and revised the draft. All authors listed have made a substantial, direct, and intellectual contribution to the work, and approved it for publication.
